# From Pathology to Intervention and Beyond. Reviewing Current Evidence for Treating Trauma-Related Disorders in Later Life

**DOI:** 10.3389/fpsyt.2022.814130

**Published:** 2022-03-01

**Authors:** Jeannette C. G. Lely, Rolf J. Kleber

**Affiliations:** ^1^Research Department, ARQ Centrum'45, Diemen, Netherlands; ^2^Department of Clinical Psychology, Utrecht University, Utrecht, Netherlands; ^3^ARQ National Psychotrauma Centre, Diemen, Netherlands

**Keywords:** older adults, PTSD, posttraumatic stress disorder, psychotherapy, recovery

## Abstract

**Background:**

An emerging body of empirical research on trauma-focused interventions for older adults experiencing symptoms of posttraumatic stress disorder or PTSD has yielded encouraging results. Nevertheless, up to date, the evidence remains scattered and is developed within rather specific groups, while studies have focused mostly on individual psychopathology, overlooking the relevance of resilience and recovering in one's social environment.

**Objective:**

This study aims at summarizing the emerging evidence on treating trauma-related disorders in older adults, followed by implications for clinical practice and future research. Specifically, the following research questions are addressed: Which factors may optimize access to intervention, what treatment benefits can be realized, and how to improve resilience by using individual as well as community-oriented approaches?

**Methods:**

A systematic literature research of intervention studies on PTSD among older adults, published between 1980 and December 2021, was expanded by cross-referencing, summarized in a narrative synthesis and supplemented with a clinical vignette reflecting qualitative outcomes.

**Results:**

Five RCTs compared varying types of trauma-focused Cognitive Behavioral Therapy with non-trauma-focused control conditions. From one of them, qualitative results were reported as well. The most recent studies reported encouraging results, confirming the suggestion that evidence-based psychotherapy for PTSD can be safely and effectively used with older adults.

**Conclusions:**

Since evidence-based psychotherapy for PTSD can be safely and effectively used with older adults, new avenues for practice and research may be found in a resilience perspective and a public mental health framework.

## Introduction

Whereas all over the world, the number of older adults is increasing (1), in research and practice regarding psychotherapy for mental health problems, including posttraumatic stress disorder or PTSD (2), older and middle-aged adults are underrepresented in comparison with younger adults (3). Although the percentage of older adults with PTSD appears to be lower than in younger adult groups (4–6), if left untreated, PTSD presents high burdens to individuals (both adults and older adults) and society (7, 8).

Disrupting experiences, however, such as exposure to domestic violence, physical attacks, sexual violence, road accidents, warfare or natural disasters may occur throughout human life and leave persisting psychological disturbances up until later life, such as PTSD. According to the current diagnostic criteria (2), there are four categories of PTSD symptoms: intrusive re-experiencing, avoidance, negative alterations in mood and cognitions, and alterations in arousal. Symptoms have to persist for more than 1 month after the precipitating (or index) event. Nevertheless, empirical findings (9–11), have stressed that most trauma survivors will not develop serious mental disorders. If they do, comorbid major depression, somatic complaints and problems in psychosocial adjustment (6, 12, 13) may accompany PTSD symptoms and predict higher PTSD symptom severity, disability days and health service utilization (8).

From a lifespan perspective, several trajectories of PTSD can be distinguished: resilience (mild and short-term symptoms), recovery (moderate symptoms gradually receding), chronicity (severe persisting symptoms) and delayed onset, meaning severe symptoms emerging in late life for the first time after an index event much earlier (9, 11). Often, symptom severity is fluctuating in response to concurrent stressors. Furthermore, first-time emergence of symptoms may occur in all stages of life following a recent traumatic event. In older adults, both full-spectrum PTSD and partial or subthreshold PTSD have been found to be threatening health and daily functioning (6).

In general population surveys, 12 months' prevalence rates of PTSD in Europe were found to vary from 0.4–6.7% (14). Prevalence rates may vary among countries with specific histories of large scale trauma, such as Germany (15) or Australia (4). Epidemiological research indicated that 90% of community-dwelling adults (55–69 years of age) were exposed to one or more types of traumatic events (16). Characteristic events in older adulthood may involve the unexpected death of a loved one, illness or accident of a loved one, personal illness or accident (16) and elder abuse (17). Notably, symptom severity was found to be highest for non-disclosed events (16). Among community-dwelling adults (≥55 years), six-month prevalence of full-spectrum PTSD was found to amount to almost 1%, whereas subthreshold PTSD amounts to 13% (6). Overall symptom severity may vary substantially; avoidance symptoms were found to distinguish between three classes of severity (18). In older adults, the long-term consequences of index events earlier in life seem to consist of decrease in intrusions and an increase in avoidance symptoms (19).

Regarding psychotherapy for patients with PTSD, the latest practice guidelines for the treatment of PTSD (20–22) recommend trauma-focused cognitive behavioral therapy (TF-CBT), such as Prolonged Exposure or PE (23), Cognitive Processing Therapy or CPT (24), Eye Movement Desensitization and Reprocessing or EMDR (25), and Narrative Exposure Therapy or NET (26), and suggest the use of Brief Eclectic Psychotherapy for PTSD or BEPP (27, 28). In these approaches, some form of trauma-focused exposure is considered to be the core mechanism of change. Hitherto, practice guidelines have not specified whether these recommendations can be applied with older adult patients with PTSD. Generalizability is not self-evident, since developmental challenges relevant to older age, such as physical and cognitive changes, might impact treatment delivery and outcome (29).

The question is whether the disorder-specific interventions provided to older adults require age-specific adjustments to show full effect (19). After all, older adults may have to cope with physical limitations, attentional or memory problems, shorter future perspectives, and loss of job (and status) due to retirement. Multimodal presentation of information material, such as offering psycho-education both in bold print and in conversation (30), flexibility in treatment pacing and scheduling appointments (31) and a life-review approach (19, 32) were proposed as useful adaptations for older adults. Likewise, a life review intervention was found to be effective in reducing depression symptoms in older adults (33). Taken together, the suggestions described might be subsumed under two broad headings: personalization of interventions (e.g., the former two adjustments) and using a developmental (life-span) perspective (the latter), which recognizes full life experience and provides coherence and acceptance.

Since some PTSD symptoms may persist after completing treatment and coexist with regaining strength and initiative (34), personal recovery (in terms of resilience and social environment) should be the overarching goal of both treatment and follow-up (35). In line with the centrality of this aim, this study focuses on how to improve recovery all along the process from access to treatment to follow-up.

This study aims at addressing those issues by updating currently available evidence on treating PTSD in older adults, focusing on recovery and addressing the following research questions: Which factors may optimize access to intervention, what treatment benefits can be realized, and how may resilience be improved by using individual as well as community-oriented approaches? To highlight the value of older PTSD patients' personal understanding of their situation and its consequences, a clinical vignette may serve as an appropriate starting point.

### Clinical Vignette, Part 1

A man, aged 63 years, married, with three children, was referred to a specialized outpatient clinic by his general practitioner with a request for additional diagnostics and a treatment advice. The GP described the patient's problems as: “nightmares and memory problems in combination with diabetes, type 2”. The patient had worked as a bus-driver, and from time to time, he had had to deal with violent passengers. He had handled these situations fairly well. The nightmares had started after the patient had collapsed during a hypoglycaemic attack. Due to impaired functioning, the patient was declared unfit for work. Concurrent with the nightmares, attention problems were observed. The GP assumed that adverse childhood experiences played a role. Before establishing a diagnosis and considering a treatment approach, age-related memory impairment had to be ruled out.

In the patient's own words: “*I was referred to this institution after a long period of stress. Actually, there was too much stress in my life. For a long time, I managed to handle it, by working hard, too hard. But when diabetes came up, I collapsed, and my old nightmares returned. In those bad dreams, I was bullied by my peers and beaten at home, as if everything happened again. I got very irritable and I had memory problems. I felt so bad…, but I thought this was due to age. After all, getting older means to get problems. But when the family doctor asked me what these bad dreams were about, he said there could be a link with what had happened to me in the past. It's kind of embarrassing to talk about personal problems with somebody other than family. In our generation, we're supposed to solve our own problems. But this just can't go on any more... You know, my wife can't handle my nightmares and my spells of anger any longer. Frankly, I never told anybody the worst details of what had happened. After all, I also feel guilty, that I couldn't stop it when it happened. Now you offer me treatment for my problems. I wonder how you can change what happened in the past. What difference will it make to talk?”*

## Methods

The literature was searched by the first author and a senior librarian. OvidSP software was used. The internet bases searched were PsychInfo and Ovid Medline. Trials published between 1980 and July 27th, 2021, were included and confirmed in December 2021; no language restrictions were applied. Search terms in titles and abstracts were: (“Posttraumatic stress disorder”) OR (“PTSD”), AND (“older adults”), AND (“treatment”). The studies had to report at least one quantitative measure of PTSD assessed both pre- and post-treatment. Searching the abstracts, the broad search term of (“treatment') was narrowed to randomized controlled trials (RCTs) reporting PTSD outcomes from individually administered trauma-focused CBT (TF-CBT) interventions compared with non-TF comparators. Finally, the results were expanded by cross-referencing and supplemented with a clinical vignette reflecting impressions from a qualitative analysis investigating older patients' personal comments during treatment.

## Results

### Study Selection

From the first set of records, 199 were deduplicated on PubMed Reference Number (PMID) and Digital Object Identifier (DOI), following well-accepted methodology (36). From the remaining 608 studies, four were redundant; 214 did not include older adults participants; 138 did not report quantitative measures of PTSD and 26 did not involve psychotherapy. A total of 226 studies was considered suitable to search the abstracts. Finally, five RCTs fulfilled the selection criteria. The quantitative results from the fifth trial were presented in two publications (37, 38).

### Study Characteristics

The selected studies were published during the past 15 years and involved 238 individuals in four countries; 117 (49.2%) engaged in an individual TF-CBT intervention. Participants varied from former political prisoners (39), military veterans (40, 41), World War II-survivors with Adverse Childhood Experiences or ACEs (42) and civilians with mixed backgrounds (37, 38). Sample sizes ranged from 11 (40) to 94 (42); the minimal age ranged from 53–63. All interventions involved some form of TF-CBT, varying from Virtual Reality Exposure Therapy (40), to Narrative Exposure (37–39), Integrative Testimonial therapy - an Internet-based form of Life Review (43) -, (42) and Prolonged Exposure (44). The comparators consisted of psycho-education (39), waiting list (42), relaxation therapy (44) and Present-Centered Therapy or PCT (37, 38, 40). Outcomes included both clinician-administered interviews and self-report measures. Most studies reported outcomes for PTSD and depression symptoms. The dropout rates varied from 0% (39) to 19.5% (44).

Although Present-Centered therapy or PCT (40, 45) was developed as a non-trauma-focused control condition, this approach has been found to be an additional safe and effective treatment approach for PTSD (37, 45). In PCT, trauma-related psycho-education concentrates on developing skills for solving problems associated with interpersonal stressors. More than providing support or strengthening avoidance, this intervention focuses on acceptance and commitment and offers active cooperation of patient and therapist. In the direct comparison, NET and PCT showed equal outcomes at follow-up, although by different routes in treatment response (37). The studies' key characteristics are presented in Table 1.

**Table 1:** Controlled trials on trauma-focused therapy with older adults: key characteristics.

**First author**	**Bichescu**	**Ready**	**Knaevelsrud**	**Thorp**	**Lely**	**Lely**
Year of publication	2007	2010	2017	2019	2019	2022
Age range	≥60	53–62	63–85	60–89	55–81	55–81
Age in Years: M (SD)	68.9 (4.4)	57.5 (3.03)	71.4 (4.7)	65 (5.7)	63.8 (6.8)	63.8 (6.8)
Number of participants	18	11	94	87	28	28
Population	Former political detainees	Military veterans	Older adults with ACE*	Military veterans	Mixed	Mixed
Country	Romania	USA	Germany	USA	Netherlands	Netherlands
Experimental condition	NET*	VRET*	ITT*	PE*	NET	NET
Control condition(s)	Psycho-education	PCT*	WLC*	RT*	PCT	PCT
Pre-to-post e.s.* PTSD (exp. condition)	d*= 3.15**		*d* = 0.84**	*d* = 0.89*		
Between-groups e.s. (post-treatment) PTSD	*d* = 1.48**	*d* = 0.24	*d* = 0.42	non-significant	*d* = −0.44*	
Between-groups e.s. (follow-up) PTSD		*d* = 0.56				
Pre-treatment to follow-up e.s. PTSD (exp. condition)						*d* = 0.54*
Pre-treatment to follow-up e.s. PTSD (control condition)						*d* = 0.34
Pre- to post-treatment e.s. depression	*d* = 0.97**		*d* = 0.36			*d* = 0.35
Pre-treatment to follow-up e.s. depression						*d* = 0.51*
Pre-treatment to follow-up e.s. general distress						*d* = 0.74**
Between-groups e.s. at post-treatment depression	*d =* 1.48**	*d* = −0.24	*d* = 0.36			
Dropout rate in experimental condition at post-treatment	0.0%	18.2%	12.8%	19.5%	16.7%	16.7%

NET, Narrative Exposure Therapy; VRET, Virtual Reality Exposure Therapy; IE, Imaginary Exposure; WLC, Waiting List Condition; PCT, Present-Centered Therapy; ITT, Integrative Testimonial Therapy (CBT with life Review via Internet); ACE, Adverse Childhood Experience(s); PE, Prolonged Exposure; RT, Relaxation Training; e.s., effect size; d, Cohen's d; Negative sign in effect size, control condition outperforms experimental condition; *p < 0.05; **p < 0.01.

## Discussion

After returning to the clinical vignette (part 2), the main findings will be summarized, followed by relevant issues for mental health professionals regarding access to treatment, treatment benefits, recovery, resilience and social environment, a public health perspective and finally strengths and limitations and implications and conclusions.

### Clinical Vignette, Part 2

“*Looking back at my therapy, I can say it really helped to talk about what had happened to me. I could tell the therapist everything. Not all my problems were solved, but an important part of my nightmares has disappeared. I should have done this much sooner. Therapy was not easy, far from. The nights after a session, I slept worse. But afterwards, it was as if I had put a step forward. The relationship with my wife also improved. When I sleep better, so does she, so together we manage better during the day. Furthermore, during therapy we discovered important moments of support…, that she really was there for me. I am grateful for that. I also discovered that I don't have to be ashamed about what had happened to me. Under the circumstances, there and then, nobody could reasonably have expected me to change things as they were. I also realize now that there is strength and endurance in me. The document drawn up at the end of therapy will help me to open up to our children, so they can understand me better. Maybe we can have a better time together in the years ahead.”*

### Main Findings

This review of empirical studies on psychotherapy for PTSD in older adults presents an emerging body of evidence. Due to varying sample sizes and age limits for inclusion, different measurement timepoints and highly diverging effect sizes, the findings were hard to align. The studies with larger samples (37, 38, 42, 44) showed more comparability and reported encouraging results, confirming the suggestion that evidence-based psychotherapy for PTSD can be safely and effectively used with older adults (46).

The present findings suggest the potential of TF-CBT to improve the mental health of older adults PTSD patients. The reported dropout rates in the current samples (0.0–19.5%) are considered small in comparison to mean dropout rates (27%) reported from a sample of clinical trials regarding PE among adults (47) or 25% in RCTs on military-related PTSD (48). When conducting empirical research on treating trauma-related disorders in later life, the challenge of recruiting participants seems to be larger than preventing dropout (40). This might be explained by symptoms of avoidance in older patients with PTSD and apprehension about adverse effects of participating in scientific research.

### Access to Treatment

Psychological treatment for older adults has been characterized by three barriers: low recognition of PTSD in primary care (8, 49), reluctance of older adults to use the services of mental health professionals to deal with their problems (50) and, until recently, a limited body of evidence concerning trauma-focused treatment for older adults. These barriers are associated with persisting and biased assumptions about older adults having insufficient flexibility to benefit from psychotherapy; an opinion derived from Sigmund Freud (51). Disconfirming these stereotypes, however, older and younger adults were found to benefit in equal measure from mental health care (52). Similarly, older and younger adults benefitted equally from high intensive EMDR treatment (53). Emerging evidence on trauma-focused exposure therapy in later life (37–40, 42, 44) has yielded encouraging findings on various clinically relevant variables. Regarding resilience measures, data from one of the selected RCTs showed that posttraumatic growth was significantly associated with treatment outcome (54). Additionally, numerous case reports (31, 46, 55–58), and uncontrolled trials (53, 54, 59–61) have suggested likewise.

The first barrier, partly caused by older adults' tendency to underreport mental health issues, presents a challenge for primary care providers. Consequently, PTSD is described as “hidden variable” in older patients' life stories (62). This tendency to underreport may be addressed by developing education material in the field of public (mental) health. By presenting the potential of trauma-focused psychotherapy with older adult PTSD patients to achieve clinically meaningful results, this review addressed the third barrier.

Access to appropriate treatment may further be improved by using age-appropriate (partly transdiagnostic) psycho-diagnostic instruments for older adults (63) and routine inclusion of hetero-anamnestic information in the intake procedure. Given the possibility of ACEs in PTSD patients' past, standard assessment should include the ACE questionnaire (World Health Organization, WHO). Since both age-related changes and PTSD symptoms can involve attentional and memory problems, cognitive functioning should be routinely assessed as well (64). Extending routine assessments with cognitive and physiological measures (blood pressure or heart rate) could provide additional evidence on risks and outcomes of psychotherapy in older adults (29, 46). Finally, e-health applications for assessment and/or intensive treatment formats (53) could bring interesting innovations and allow access to additional groups of patients.

### Treatment Benefits

The vignette illustrates the possibility of regaining stability and initiative notwithstanding residual symptoms. Traumatic experiences are followed by a process of coping or adaptation (9, 65). After such experiences, one has to adapt to a world that has changed forever and is left to rebuild one's view on oneself, one's environment and one's future. People will look for explanations of what happened to them, trying to comprehend the implications in the light of their own personal existence. Finding a meaning for the experience gives an individual a sense of coherence (66, 67). This process of making sense is connected with emotions. One goes through the pain of the aftermath of the experience and may be confronted with many, often conflicting feelings and thoughts, while not avoiding or suppressing them. Eventually, people can actually thrive in the aftermath of the traumatic event (68). Coherence may bring about a sense of control. Posttraumatic coping will improve when one has the feeling of control over certain situations, however small this control may be (69). By learning to leave the past in the past, one can get more grip on the present. “*I am stronger than my ghosts*!” (70).

As for the “years ahead”, expanding life expectancy implies that improved functioning following treatment offers a perspective on potentially many more years of a satisfactory quality of life. This awareness may diminish feelings of regret about the many years spent coping with painful symptoms and memories. Grief about missed opportunities or deteriorated relationships might require attention. Renewed self-esteem may enable patients to aim at overcoming long-standing estrangement with relatives. In these years, new understanding between parents and children or grandchildren may develop, potentially correcting or mitigating previous intergenerational transmission of maladaptive interaction patterns (71).

### Recovery

Applying evidence-based treatment approaches with older PTSD patients can be considered good clinical practice, potentially protecting against further demoralization or disability. Since both trauma-focused and a (trauma-related) present-centered approach (PCT) have been found safe and effective, shared decision making and fine-tuning treatment plans to patients' needs and priorities is possible and may protect against feelings of helplessness which often accompany PTSD, and improve the process of adjustment. Habituation to adverse stimuli, differentiating the past from the present, cognitive reprocessing, meaning making and improved coherence are considered the protecting factors in all treatments. The same accounts for social recognition of posttraumatic suffering (72). Moreover, in case of co-occurring somatic treatment, e.g. by a cardiologist or neurologist, interdisciplinary collaboration will be required. Besides, informing and co-operating with partners, children or caregivers will be essential to ensure safety and continuity during treatment. Finally, to ensure and enhance high-quality treatment, continuous efforts on treatment adherence, outcome data collection and dissemination of research findings are recommended.

Box 1 **Overview of recommendations.**

**Apply evidence-based interventions of controlled quality**

- Use Trauma-focused CBT for PTSD1. Prolonged exposure (PE)

2. Internet-based Integrative Testimonial Therapy (ITT)
3. Virtual Reality Exposure Therapy (VRET)
4. Narrative Exposure Therapy (NET)
5. Eye Movement Desensitization and Reprocessing (EMDR)- Consider Present-centered therapy as viable alternative
6. Present Centered Therapy (PCT)- Control treatment quality
7. Adhere to treatment protocols
8. Describe (reasons of) deviations from protocol
9. Collect data from the provided treatments
10. Aim at long-term follow-up assessments**Adapt treatment procedures age-appropriately**

- Use tailor-made treatment plans

1. Win patients' confidence
2. Offer psycho-education
3. Use multi-modal information material
4. Use age-appropriate diagnostic instruments
5. Take into account sensory and functional difficulties
6. Use adjunct interventions in case of cognitive limitations
7. Address transport difficulties
8. Use flexible treatment pacing
9. Provide structure and repetition
10. Report all adaptations and its reasons- Include a lifespan perspective
11. Improve coherence by including a biographic overview
12. Offer social recognition by validating life experiences**Adopt a wide scope**

- Address personal resilience factors

1.Take into account patients' priorities by reaching shared decisions
2. Assess strengths and vulnerabilities (transdiagnostic assessments)
3. Inform co-treating specialists, such as cardiologists or neurologists
4. Aim at relapse prevention- Mind the social environment
5. Use hetero-anamnestic assessments
6. Include a trusted family member or friend in psycho-education
7. Educate colleagues in primary health care and social services
8. Organize adjunct systemic interventions if necessary
9. Be aware of intergenerational transmission of trauma-related problems
10. Discuss plans for the future

- Accept a role in public mental health efforts

11. Support dissemination of research findings
12. Provide information on risk factors for developing PTSD in later life

### Resilience and Social Environment

Posttraumatic mental health problems have been acknowledged as serious conditions. Trauma research and treatment, however, have been dominated by focusing on psychopathology and individual symptom**s**, preferably measured with clinician-administered assessments. With older cohorts, however, additional attention is necessary for systemic issues, idioms of distress, the social environment and resilience factors. After all, recovery from PTSD may allow for resuming one's development and find renewed strength and posttraumatic growth, even following multiple instances of abuse in late life (17). Personally reflecting on one's recovery may enhance self-efficacy (73) and coherence (66). Understanding the patient's personal experiences during psychotherapy may be addressed by using outcome monitoring, e.g. by adding personal interviews to the measurements, preferably in structured, systematic ways (70).

### A Public Health Perspective

Adopting a public health perspective allows for joining age-specific and disorder-specific aspects. This outlook may include paying attention to the context of mental disturbances and understanding health, illness and distress in terms of the individual's (social, political and cultural) environment. Doing so, mental health professionals should be watchful not to overlook older patients' potential economic difficulties (on an individual or family level). Informing social care professionals might be useful to allow patients access to - and completion of - treatment. This contextual approach may enhance secondary prevention and extend the impact of interventions (65), protecting against symptom relapse and social isolation. In addition, this perspective especially requires focusing on the wider setting in which health after trauma may be threatened and on necessary protecting interventions. Shifting the focus from symptoms to resilience variables (such as self-efficacy and coherence) and from the individual patients to their family as well as community can serve this goal. This focus may imply a completed trauma-focused treatment to be followed by a (short) systemic intervention, for instance to guide patients in disclosing new, possibly disturbing, information about their past to their family members, or in trying to change estranged relationships. The same accounts for grief work in case of traumatic losses. Regarding prevention, professional risks on developing PTSD (aging combat veterans, police personnel or firefighters) might be diminished by providing retirement-related psycho-education (74). An overview of all recommendations mentioned is provided in Box 1.

## Strengths and Limitations

The present study summarizes recent developments in the highly relevant field of treating PTSD in older adults. Its strengths include a systematic literature selection and enrichment of the presentation with patient-reported outcomes derived from one of the described studies. Some limitations should be mentioned. Different age limits were used for inclusion, limiting the comparability of the studies. The resulting samples did not allow inclusion of sufficient older-old participants to reflect current demographic developments (75). Furthermore, it should be noted that the research examining mainstream interventions for PTSD in older adults is limited. The present results need to be interpreted together with findings from future studies, that will have preferably larger sample sizes and extended age ranges. The implementation of the suggested avenues and perspectives may inspire new ways of interdisciplinary and transmural collaboration, guiding future research.

## Implications and Conclusions

Returning to the research questions, access to treatment (a), treatment benefits (b) and optimizing recovery (c), were addressed successively. Timely detection of PTSD-symptoms in primary care allows for appropriate referrals or treatment advice. Available evidence regarding trauma-focused exposure treatment for older adults with posttraumatic stress symptoms (37–40, 42, 44) has yielded encouraging results. Trauma-focused and present-centered interventions have been found to be safe, acceptable and effective for this population, although extended research (including older participants, or PTSD patients from other backgrounds) would be highly desirable. Disconfirming long-standing stereotypes regarding older adults' capacity to change, treatment response from psychotherapy has been found to be invariant with age. Understanding the patient's personal experiences during psychotherapy is addressed by including outcome monitoring into therapeutic procedures. Widening the scope of treating older adults with trauma-related disorders may inspire new initiatives and research avenues.

Summarizing, these implications allow for the following conclusions. First: whereas PTSD has been described as a hidden variable in the lives of older adults, evidence-based interventions and a community perspective may appeal to hidden strengths and resources. Second: psychotherapy for trauma-related disorders in later life may be meaningful for years ahead. Third: pessimism concerning the treatment of older adults with PTSD is unfounded and using an individual pathology-oriented perspective too narrow. By overcoming these mindsets, treating trauma-related disorders in later life is coming of age.

## Author Contributions

JL and RK jointly designed and wrote this paper. Both authors contributed to the article and approved the submitted version.

## Conflict of Interest

The authors declare that the research was conducted in the absence of any commercial or financial relationships that could be construed as a potential conflict of interest.

## Publisher's Note

All claims expressed in this article are solely those of the authors and do not necessarily represent those of their affiliated organizations, or those of the publisher, the editors and the reviewers. Any product that may be evaluated in this article, or claim that may be made by its manufacturer, is not guaranteed or endorsed by the publisher.
